# Application of the Chinese Version of the Montreal Cognitive Assessment-Basic for Assessing Mild Cognitive Impairment in Parkinson’s Disease

**DOI:** 10.3390/brainsci11121575

**Published:** 2021-11-28

**Authors:** Qian Xu, Mengxi Zhou, Chunyan Jiang, Li Wu, Qing He, Lei Zhao, Yourong Dong, Jianren Liu, Wei Chen

**Affiliations:** Department of Neurology, Shanghai Ninth People’s Hospital, Shanghai Jiao Tong University School of Medicine, Shanghai 200011, China; xuqian1969@sjtu.edu.cn (Q.X.); mency0510@163.com (M.Z.); xiaopangpang0321@163.com (C.J.); breezy51@163.com (L.W.); heqing19830531@163.com (Q.H.); caoyutian1983@163.com (L.Z.); dongyourong@126.com (Y.D.); liujr021@vip.163.com (J.L.)

**Keywords:** Parkinson’s disease, mild cognitive impairment, Montreal Cognitive Assessment-Basic, rapid eye movement sleep behavior disorder, non-motor symptom

## Abstract

Mild cognitive impairment (MCI) is a common and pivotal non-motor symptom in Parkinson’s disease (PD). It is necessary to use the appropriate tools to characterize the cognitive profiles and identify the subjects at risk of MCI in clinical practice. A cohort of 207 non-demented patients with PD and 52 age- and gender-matched cognitively normal controls (NCs) underwent the Chinese Version of Montreal Cognitive Assessment-Basic (MoCA-BC) evaluation. Patients with PD also received detailed motor and non-motor evaluation by serial scales. Cognitive profiles were investigated in patients with PD-MCI, relative to patients with normal cognition (PD-NC) and cognitively NCs. In addition, differences in demography, major motor and non-motor symptoms were compared between patients with PD-MCI and PD-NC. There were 70 patients with PD-MCI, occupying 33.8% of the total patients. Patients with PD-MCI had impairment in multiple cognitive domains, especially in executive function, memory and visuospatial function on MoCA-BC, relative to cognitively NCs or PD-NC. Compared with PD-NC patients, PD-MCI patients were older (*p* = 0.002) and had a later onset age (*p* = 0.007) and higher score of the Unified Parkinson’s Disease Rating Scale (UPDRS) part III (*p* = 0.001). The positive rate of clinical possible rapid eye movement sleep behavior disorder (cpRBD) in the PD-MCI group was significantly increased relative to the PD-NC group (*p* = 0.003). Multivariate logistic analysis showed that older age (OR = 1.06; *p* = 0.012), higher score of UPDRS-III (OR = 1.03; *p* = 0.018) and the presence of cpRBD (OR = 2.10; *p* = 0.037) were independently associated factors of MCI in patients with PD. In conclusion, executive function, memory and visuospatial function are the main impaired cognitive profiles in PD-MCI via MoCA-BC. Aging, motor severity and RBD may be independently related factors of MCI in PD.

## 1. Introduction

As a common non-motor symptom in Parkinson’s disease (PD), cognitive impairment contributes to impaired health-related quality of life to the patients and increased economic burden to society [1,2]. In most cases, mild cognitive impairment (MCI) in PD is a primary indicator of the transition between normal cognition and PD dementia (PDD). It is common in non-demented PD patients with a prevalence rate ranging from 18.9% to 38.2% [3]. Increasing age, late-onset disease, lower education level and more severe motor symptoms have been associated with PD-MCI [3,4]. Timely identification of PD-MCI is important as it is a strong predictor for the progression to PDD. Because of the important clinical implications of PD-MCI, it is necessary to use appropriate tools to assess MCI and identify the subjects at risk of PD-MCI in clinical practice.

The accurate diagnosis of PD-MCI needs comprehensive assessment with detailed neuropsychological scales, as the Movement Disorder Society Task Force proposed [4]. However, these comprehensive tests may not always be practical or available. Abbreviated assessments, such as scales with global cognition abilities, are also needed in clinical practice. Although the Montreal Cognitive Assessment (MoCA) is recommended for assessment of global cognitive performance in PD [5], it is not applicable for populations with low education levels, as its several sub-tests incorporate tasks that may be compromised by the patient’s level of education or literacy. The MoCA-Basic (MoCA-B) is a revised version of MoCA test, which consists of nine parts: executive function, language, orientation, calculation, abstraction, delayed recall, visual perception, naming and attention [5]. The Chinese version of the MoCA-B (MoCA-BC) was translated from the original English version and validated to be a reliable tool in screening MCI across all educational levels in Chinese elderly adults, and the corresponding cut-off values of MCI were defined [6]. To our knowledge, it has not been applied in Chinese patients with PD.

Therefore, the present study aimed to characterize the cognitive profiles of patients with PD-MCI via MoCA-BC, compared with PD with normal cognition (PD-NC) and cognitively normal controls (NCs). In addition, motor and non-motor correlations with PD-MCI were also explored among patients in this cohort.

## 2. Materials and Methods

### 2.1. Subjects

From January 2017 to December 2020, 223 consecutive PD patients who met the Movement Disorder Society (MDS) criteria [7] were registered in this cross-sectional study from the Department of Neurology, Shanghai Ninth People’s Hospital, Shanghai Jiao Tong University School of Medicine. Patients with diseases that may impair cognitive evaluation, such as stroke, hydrocephalus, brain tumor and epilepsy, were excluded. A total of 52 age- and gender-matched cognitively normal controls enrolled from the Chinese validation study of MoCA-BC were also included [8]. This study was approved by the Medical Ethics Committee of Shanghai Ninth People’s Hospital, and informed consent was obtained from all participants.

### 2.2. General Clinical Evaluation

We collected demographic data such as age, gender, level of education, age of onset, disease duration, family history and medication history. Levodopa equivalent daily dose (LEDD) was calculated as reported [8]. Motor severity was assessed by the modified Hoehn and Yahr (H&Y) stage [9] and Unified Parkinson’s Disease Rating Scale part III (UPDRS-III) [10] during the “on” state. Motor subtype (tremor-dominant, akinetic-rigid, mixed) was further defined according to the report from Kang et al. [11]. Freezing of gait (FOG) was considered when subjects had a score of more than one on item 3 of the Freezing of Gait questionnaire (FOG-Q) [12]. Olfactory function was assessed by SS-16 and a score of less than 8.3 was defined as hyposmia [13]. The Rapid Eye Movement (REM) Sleep Behavior Disorder Screening Questionnaire (RBDSQ) was used to screen clinical possible rapid eye movement sleep behavior disorder (cpRBD), and a score of more than 6 was determined as cpRBD [14]. The severity of depressive symptoms was assessed by the 17-item Hamilton Rating Scale for Depression (HAMD-17) and a score of more than 8 was regarded as depression [15]. The Scales for Outcomes in PD Autonomic Dysfunction (SCOPA-AUT) was used as a measurement of autonomic disfunction [16]. Constipation was defined according to item 5 in SCOPA-AUT [17].

### 2.3. Cognitive Evaluation

For PD patients and cognitively NCs, global cognitive function was assessed by the Chinese version of MMSE and MoCA-BC. Cognitively NCs underwent detailed neuropsychological tests to ensure normal cognitive function, as a previous study reported [6]. Dementia patients were excluded based on their MMSE score: below 17 for subjects without formal education; below 20 for individuals that received 6 years of schooling or less (primary school education) and below 24 for individuals that received more than 6 years of schooling (junior and above) [18].

MoCA-BC is a 30-point test that assesses nine cognitive domains (executive function, language, orientation, calculation, conceptual thinking, memory, visual perception, naming and attention) and is freely available for clinical use (www.mocatest.org, visit Basic section, accessed on 1 August 2015). According to the first validation study in China from Qihao Guo, the cut-off scores of the MoCA-BC for MCI detection were 19 in the low-level education group (≤6 years), 22 in the mid-level education group (7–12 years) and 24 in the high-level education group (>12 years), respectively [6].

**Figure 1:**
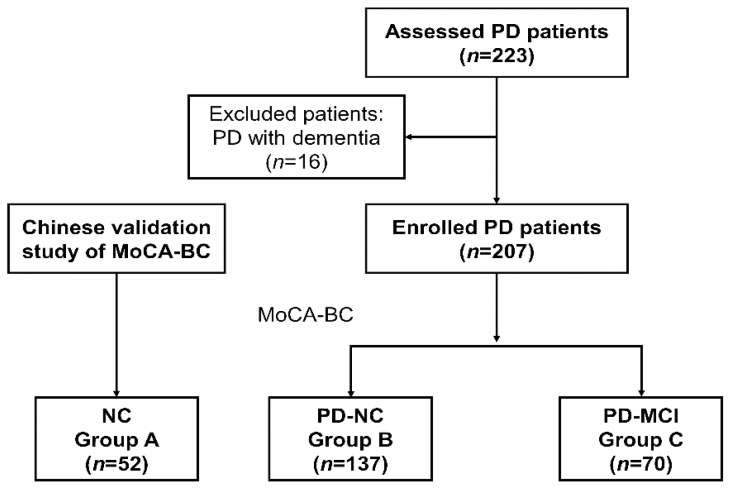
The flow chart diagram of the study. Abbreviations: PD, Parkinson’s disease; NC, normal control; PD-NC, Parkinson’s disease with normal cognition; PD-MCI, Parkinson’s disease with mild cognitive impairment; MoCA-BC, The Chinese version of the Montreal Cognitive Assessment-Basic.

Two hundred and twenty-three patients with PD completed all the clinical evaluation; 16 patients were defined as PD with dementia (PDD) and were excluded from the present study. Finally, 70 patients with PD-MCI, 137 patients with PD-NC and 52 cognitively NCs were recruited for the final analysis (Figure 1).

### 2.4. Statistical Analysis

SPSS version 23.0 (IBM Corporation, Armonk, NY, USA) was used for statistical analysis. Continuous variables are expressed as the means ± SD or medians (interquartile ranges (IQR)); categorical variables are expressed as frequencies and percentages. Comparisons of means between the two groups were performed using the independent t test or the Mann-Whitney U test as appropriate. The chi-square or Fisher exact test was used for comparing proportions. For the total scores and sub-score in each cognitive domain among PD-MCI, PD-NC and cognitively NCs, we analyzed the continuous variables by one-way analysis of variance (ANOVA) or non-parametric Kruskal-Wallis tests, depending on whether the data were normally distributed or not. For clinical characteristics with a significant *p*-value for the global test, each of the three groups was separately compared to each other. The *p*-values for these three group comparisons were adjusted using the Bonferroni method. The independent factors affecting MCI were analyzed by a multivariate logistic regression model. The test level (α) was 0.05.

## 3. Results

### 3.1. Demographic Data

The general characteristics of the patients with PD are shown in Table 1. The mean age of the patients was 66.1 ± 7.6 years and the mean age of onset was 63.0 ± 8.1 years. Thirty-six patients (17.4%) reported family history of PD or tremor. From the cohort, 190 (91.8%) subjects had early stage disease (H&Y 1–2.5), whereas 17 (8.2%) patients had moderate- to late-stage disease (H&Y 3–4). Seventy-four patients (35.7%) were de novo PD. Age (*p* = 0.462), gender (*p* = 0.097) and education level (*p* = 0.697) were consistent between PD and the cognitively NC group (Table 1).

There were 70 subjects with PD-MCI, accounting for 33.8% of the total PD patients. Compared with the PD-NC group, patients with PD-MCI were older (*p* = 0.002) and had a later age of onset (*p* = 0.007). There was a trend that males had more cognitive impairments, but with no statistical significance (*p* = 0.086). No significant difference was found for disease duration, family history, education level or drug usage and dosage (Table 1 and Table S1).

### 3.2. Characteristics of Cognitive Impairment in PD Patients with MCI

**Figure 2:**
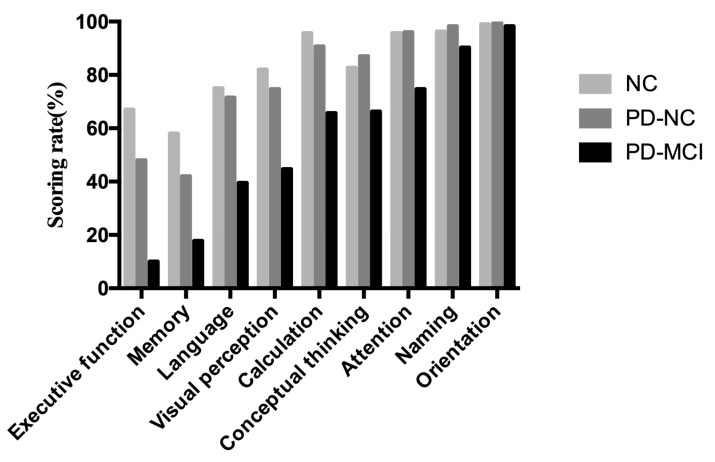
Scoring rate of each cognitive domain from MoCA-BC in cognitively NCs, patients with PD-NC and PD-MCI. Abbreviations: PD, Parkinson’s disease; NC, normal control; PD-NC, Parkinson’s disease with normal cognition; PD-MCI, Parkinson’s disease with mild cognitive impairment; scoring rate refers to the mean value of actual score divided by corresponding sub-item maximum score.

The total scores and sub-score in each cognitive domain among PD-MCI, PD-NC and cognitively NCs are shown in Table 2. There were significant differences in nearly each domain of cognition except for orientation on MoCA-BC (Table 2). A histogram was delineated to demonstrate the mean scoring rate of each cognitive domain in MoCA-BC among the three groups (Figure 2). It revealed that executive function (10%), memory (17.8%), language (39.5%) and visuospatial function (44.7%) were the four primarily affected cognitive domains in patients with PD-MCI.

### 3.3. Clinical Characteristics Associated with MCI in Patients with PD

We found that PD-MCI patients were older (*p* = 0.002, Table 1) and had more severe motor symptoms, as revealed by the UPDRS-III score (*p* < 0.01, Table 3), compared with the PD-NC group. The positive rate of cpRBD in PD-MCI patients was higher than that of PD-NC patients (*p* = 0.003, Table 3). There was no significant difference in motor subtype, laterality, positive rate of FOG or motor complications between the two groups. The other non-motor symptoms, such as hyposmia, autonomic dysfunction, depression and hallucination, also was comparable between the two groups.

We further investigated the independent factors associated with MCI in PD patients. By univariate logistic regression analyses, we found old age (OR = 1.07; 95% CI: 1.02–1.12; *p* = 0.003), high score of UPDRS-III (OR = 1.04; 95% CI: 1.01–1.06; *p* = 0.003) and cpRBD (OR = 2.5; 95% CI: 1.37–4.58; *p* = 0.003) were associated with MCI in PD. After adjusting for the potential confounding factors such as gender, hyposmia and constipation, the above three factors, old age (OR = 1.06; 95% CI: 1.01–1.12; *p* = 0.012), high score of UPDRS-III (OR = 1.03; 95% CI: 1.01–1.06; *p* = 0.018) and the presence of cpRBD (OR = 2.1; 95% CI: 1.05–4.22; *p* = 0.037), were still independent factors of PD-MCI (Table 4), as demonstrated in the multivariate logistic regression analysis.

## 4. Discussion

This cross-sectional study was the first application of MoCA-BC for assessing MCI in a Chinese cohort of PD patients. Our results demonstrated that PD patients with MCI had impairments in multiple cognitive domains except for orientation; old age, severe motor symptoms and positive cpRBD were associated with MCI in PD patients.

We found that 33.8% of PD patients had MCI, which was consistent with previous reports with the MCI prevalence ranging from 18.9% to 38.2% [3]. In the current study, the full spectrum of cognitive impairments was observed in PD with MCI. The language domain in MoCA-BC refers to the verbal fluency test, which is considered to be a type of executive function. Therefore, executive function, memory and visuospatial function were found to be the three most impaired cognitive domains, whilst orientation domain was preserved with respect to scoring rate on MoCA-BC. As previous studies reported, the pattern of cognitive impairment in PD is variable [19]. Executive deficits usually appear as the most frequent type of cognitive impairment in early PD [20], while up to 40% of patients exhibit deficits in other cognitive domains such as visuospatial skills and memory [21]. Baseline neuropsychological profile in early-stage PD may predict the risk of developing dementia. Factor analysis in a previous study [22] demonstrated that the composite of executive, visuospatial and verbal memory deficits was associated with a higher risk of dementia conversion. Among them, frontal/executive dysfunction contributed most to the occurrence of PDD [22]. The consistency with previous results allows us to confirm the value of MoCA-BC in assessing MCI in Chinese PD patients.

Consistent with previous studies, we found that both age and the severity of motor symptoms were independent factors associated with PD-MCI. Age was known to be the strongest clinical predictor of cognitive impairment, both in the general population and in patients with PD [23,24,25]. Patients with severer motor symptoms were more likely to be classified as cognitively impaired than those with less severe motor symptoms [26,27]. When combining the effects of age and the severity of extrapyramidal signs, risk of incident dementia in PD was significantly increased [26]. In addition to motor symptoms, we also found that one non-motor symptom, RBD, was closely related to MCI in PD, which was also in line with previous cross-sectional and longitudinal studies [28]. Natalia Jozwiak et al. found that PD patients with RBD had poorer performance on cognitive tests measuring attention, executive functions, language, memory and visuospatial abilities [29]. Longitudinal studies demonstrated that the presence of RBD in established PD could increase the risk of dementia [24]. It has been hypothesized that the presence of clinical RBD in PD could be accounted for by progressive neocortical, limbic, cortical and thalamic cholinergic denervation [30]. Consequently, PD patients with RBD should be carefully screened clinically for the presence of cognitive impairment.

Some limitations should be noted in the present study. First, the cut-off value of MoCA-BC to define MCI comes from a recent validated study among elderly adults in Shanghai, China. Future studies are needed with detailed neuropsychological tests both in PD patients and NCs to define the optimal cut-off value of MoCA-BC in the detection of PD-MCI. Second, the diagnosis of cpRBD was based on RBDSQ, and was not confirmed by polysomnography (PSG). However, the overall cognitive characteristics and the clinical correlates of MCI in PD patients were consistent with previous findings, suggesting that the sample is representative. Follow-up studies are warranted in the future to investigate the predictors of conversion from PD-MCI to PDD.

## 5. Conclusions

By utilizing of MoCA-BC assessment in patients with PD, the current study demonstrates that PD patients with MCI have impairments in multiple cognitive domains, especially in executive function, memory and visuospatial function. Patients with increasing age, more severe motor symptoms and the presence of RBD seem to be more susceptible to MCI in PD. Therefore, we advocate the application of MoCA-BC as a global cognitive assessing tool for PD patients in clinical practice.

## Figures and Tables

**Table 1:** Demographic data in patients with PD and the cognitively normal controls.

Items	Total PD	NC (Group A)	PD-NC (Group B)	PD-MCI (Group C)	*p* Value	
					(B + C vs. A)	(C vs. B)
Number, *n*	207	52	137	70		
Age (years)	66.1 ± 7.6	65.8 ± 6.9	64.9 ± 7.7	68.3 ± 6.9	0.462	0.002 **
Male, *n* (%)	122 (58.9%)	28 (53.8%)	75 (54.7%)	47 (67.1%)	0.097	0.086
Onset age (years)	63.0 ± 8.1	NA	61.9 ± 8.4	65.0 ± 6.9	NA	0.007 **
Disease duration(years)	3.2 ± 3.6	NA	3.1 ± 3.3	3.3 ± 4.3	NA	0.642
Family history, *n* (%)	36 (17.4%)	NA	23 (16.8%)	13 (18.6%)	NA	0.749
Education level, *n* (%)					0.697	0.432
≤6 years	25 (12.1%)	7 (13.5%)	14 (10.2%)	11 (15.7%)		
7–12 years	140 (67.6%)	32 (61.5%)	93 (67.9%)	47 (67.1%)		
>12 years	42 (20.3%)	13 (25.0%)	30 (21.9%)	12 (17.2%)		

Abbreviations: PD, Parkinson’s disease; NC, normal controls; PD-NC, Parkinson’s disease with normal cognition; PD-MCI, Parkinson’s disease with mild cognitive impairment. NA, Not applicable. ** *p* < 0.01.

**Table 2:** Cognitive profiles in patients with PD-MCI on MoCA-BC.

Items (Maximum Score)	NC (Group A)	PD-NC (Group B)	PD-MCI (Group C)	*p* Value (C vs. B; C vs. A; B vs. A)
Executive function (1)	0.67 ± 0.47	0.48 ± 0.50	0.10 ± 0.30	<0.001 **; <0.001 **; 0.052
Language (2)	1.50 ± 0.50	1.43 ± 0.57	0.79 ± 0.66	<0.001 **; <0.001 **; 1.000
Orientation (6)	5.94 ± 0.24	5.97 ± 0.17	5.90 ± 0.30	0.072; 1.000; 1.000
Calculation (3)	2.87 ± 0.40	2.72 ± 0.60	1.97 ± 1.08	<0.001 **; <0.001 **; 0.634
Conceptual thinking (3)	2.48 ± 0.70	2.61 ± 0.71	1.99 ± 1.00	<0.001 **; 0.020 *; 0.520
Memory (5)	2.90 ± 1.35	2.10 ± 1.55	0.89 ± 1.17	<0.001 **; <0.001 **; 0.005 **
Visual perception (3)	2.46 ± 0.58	2.24 ± 0.73	1.34 ± 0.96	<0.001 **; <0.001 **; 0.322
Naming (4)	3.85 ± 0.36	3.93 ± 0.28	3.61 ± 0.62	<0.001 **; 0.030 *; 0.333
Attention (3)	2.87 ± 0.40	2.88 ± 0.46	2.24 ± 1.11	<0.001 **; <0.001 **; 1.000
Total (30)	25.58 ± 2.30	24.39 ± 2.33	17.96 ± 3.89	<0.001 **; <0.001 **; 0.060

Abbreviations: PD, Parkinson’s disease; NC, normal controls; PD-NC, Parkinson’s disease with normal cognition; PD-MCI, Parkinson’s disease with mild cognitive impairment. * *p* < 0.05; ** *p* < 0.01; Scoring rate refers to the mean value of actual score divided by the total score of the corresponding item.

**Table 3:** Clinical features in patients with PD-MCI.

Items	Total PD	PD-NC	PD-MCI	*p* Value
Motor symptom				
UPDRS-III	18.0 (10.0, 28.0)	16.0 (9.0, 26.5)	23.0 (14.8, 30.8)	0.001 **
Motor subtype, *n* (%)				0.501
Tremor-dominant (TD)	73 (35.3%)	45 (32.8%)	28 (40.0%)	
Akinetic-rigid(A-R)	111 (53.6%)	75 (54.8%)	36 (51.4%)	
Mixed	23 (11.1%)	17 (12.4%)	6 (8.6%)	
Laterality, *n* (%)				0.400
Left	85 (41.1%)	54 (39.4%)	31 (44.3%)	
Right	72 (34.8%)	52 (38.0%)	20 (28.6%)	
Bilateral	50 (24.1%)	31 (22.6%)	19 (27.1%)	
FOG+, *n* (%)	46 (22.2%)	29 (21.2%)	17 (24.3%)	0.610
Non-motor symptoms				
Hyposmia, *n* (%)	133 (64.3%)	82 (59.9%)	51 (72.9%)	0.065
cpRBD, *n* (%)	69 (33.3%)	36 (26.3%)	33 (47.1%)	0.003 **
Constipation, *n* (%)	103 (49.8%)	62 (45.3%)	41 (58.6%)	0.070
Depression, *n* (%)	61 (29.5%)	36 (26.3%)	25 (35.7%)	0.159
Hallucination, *n* (%)	11 (5.3%)	7 (5.1%)	4 (5.7%)	1.000
SCOPA-AUT	10.0 (6.00, 16.0)	9.0 (6.0, 15.0)	11.0 (6.0, 17.0)	0.214
Motor complications				
Wearing off, *n* (%)	33 (15.9%)	22 (16.1%)	11 (15.7%)	0.949
Dyskinesia, *n* (%)	5 (2.4%)	5 (3.6%)	0 (0.0%)	0.254

Abbreviations: PD, Parkinson’s disease; PD-NC, Parkinson’s disease with normal cognition; PD-MCI, Parkinson’s disease with mild cognitive impairment; UPDRS, Unified Parkinson’s Disease Rating Scale; TD, Tremor dominant; A-R, Akinetic-rigid; FOG, freezing of gait; +, positive; cpRBD, clinical possible rapid eye movement sleep behavior disorder; SCOPA-AUT, The Scale for Outcomes in PD autonomic dysfunction. ** *p* < 0.01.

**Table 4:** Binary logistic regression analyses for the independent factors associated with PD-MCI.

Variables	Model 1 Univariate		Model 2 Multivariate	
	OR (95% CI)	*p* Value	OR (95% CI)	*p* Value
Age (years)	1.07 (1.02–1.12)	0.003 **	1.06 (1.01–1.12)	0.012 *
Male	0.59 (0.32–1.08)	0.088	0.85 (0.44–1.64)	0.635
UPDRS-III	1.04 (1.01–1.06)	0.003 **	1.03 (1.01–1.06)	0.018 *
Hyposmia	1.80 (0.96–3.37)	0.066	1.29 (0.65–2.57)	0.465
Constipation	1.71 (0.96–3.06)	0.071	0.88 (0.44–1.76)	0.716
cpRBD	2.50 (1.37–4.58)	0.003 **	2.10 (1.05–4.22)	0.037 *

Abbreviations: PD-MCI, Parkinson’s disease with mild cognitive impairment; UPDRS, Unified Parkinson’s Disease Rating Scale; cpRBD, clinical possible rapid eye movement sleep behavior disorder; OR, odds ratio; CI, confidential interval; * *p* < 0.05; ** *p* < 0.01.

## Data Availability

The datasets used and analyzed during the current study are available from the corresponding author on reasonable request.

## Supplementary Materials

The following are available online at https://www.mdpi.com/article/10.3390/brainsci11121575/s1, Table S1: Drug usage of enrolled patients with PD.

## References

[1] Aarsland D., Batzu L., Halliday G.M., Geurtsen G.J., Ballard C., Chaudhuri K.R., Weintraub D. (2021). Parkinson Disease-Associated Cognitive Impairment. Nat. Rev. Dis. Primers.

[2] Lawson R.A., Yarnall A.J., Duncan G.W., Breen D.P., Khoo T.K., Williams-Gray C.H., Barker R.A., Collerton D., Taylor J.-P., Burn D.J. (2016). Cognitive Decline and Quality of Life in Incident Parkinson’s Disease: The Role of Attention. Parkinsonism Relat. Disord..

[3] Litvan I., Aarsland D., Adler C.H., Goldman J.G., Kulisevsky J., Mollenhauer B., Rodriguez-Oroz M.C., Tröster A.I., Weintraub D. (2011). MDS Task Force on Mild Cognitive Impairment in Parkinson’s Disease: Critical Review of PD-MCI. Mov. Disord..

[4] Litvan I., Goldman J.G., Tröster A.I., Schmand B.A., Weintraub D., Petersen R.C., Mollenhauer B., Adler C.H., Marder K., Williams-Gray C.H. (2012). Diagnostic Criteria for Mild Cognitive Impairment in Parkinson’s Disease: Movement Disorder Society Task Force Guidelines. Mov. Disord..

[5] Skorvanek M., Goldman J.G., Jahanshahi M., Marras C., Rektorova I., Schmand B., van Duijn E., Goetz C.G., Weintraub D., Stebbins G.T. (2018). Global Scales for Cognitive Screening in Parkinson’s Disease: Critique and Recommendations. Mov. Disord..

[6] Chen K.-L., Xu Y., Chu A.-Q., Ding D., Liang X.-N., Nasreddine Z.S., Dong Q., Hong Z., Zhao Q.-H., Guo Q.-H. (2016). Validation of the Chinese Version of Montreal Cognitive Assessment Basic for Screening Mild Cognitive Impairment. J. Am. Geriatr. Soc..

[7] Postuma R.B., Berg D., Stern M., Poewe W., Olanow C.W., Oertel W., Obeso J., Marek K., Litvan I., Lang A.E. (2015). MDS Clinical Diagnostic Criteria for Parkinson’s Disease. Mov. Disord..

[8] Tomlinson C.L., Stowe R., Patel S., Rick C., Gray R., Clarke C.E. (2010). Systematic Review of Levodopa Dose Equivalency Reporting in Parkinson’s Disease. Mov. Disord..

[9] Hoehn M.M., Yahr M.D. (1967). Parkinsonism: Onset, Progression and Mortality. Neurology.

[10] Richards M., Marder K., Cote L., Mayeux R. (1994). Interrater Reliability of the Unified Parkinson’s Disease Rating Scale Motor Examination. Mov. Disord..

[11] Kang G.A., Bronstein J.M., Masterman D.L., Redelings M., Crum J.A., Ritz B. (2005). Clinical Characteristics in Early Parkinson’s Disease in a Central California Population-Based Study. Mov. Disord..

[12] Giladi N., Shabtai H., Simon E., Biran S., Tal J., Korczyn A. (2000). Construction of Freezing of Gait Questionnaire for Patients with Parkinsonism. Parkinsonism Relat. Disord..

[13] Chen W., Chen S., Kang W.-Y., Li B., Xu Z.-M., Xiao Q., Liu J., Wang Y., Wang G., Chen S.-D. (2012). Application of Odor Identification Test in Parkinson’s Disease in China: A Matched Case-Control Study. J. Neurol. Sci..

[14] Nomura T., Inoue Y., Kagimura T., Uemura Y., Nakashima K. (2011). Utility of the REM Sleep Behavior Disorder Screening Questionnaire (RBDSQ) in Parkinson’s Disease Patients. Sleep Med..

[15] Hamilton M. (1960). A Rating Scale for Depression. J. Neurol. Neurosurg. Psychiatry.

[16] Verbaan D., Marinus J., Visser M., van Rooden S.M., Stiggelbout A.M., van Hilten J.J. (2007). Patient-Reported Autonomic Symptoms in Parkinson Disease. Neurology.

[17] Leta V., Urso D., Batzu L., Weintraub D., Titova N., Aarsland D., Martinez-Martin P., Borghammer P., van Wamelen D.J., Yousaf T. (2021). Constipation Is Associated with Development of Cognitive Impairment in de Novo Parkinson’s Disease: A Longitudinal Analysis of Two International Cohorts. J. Parkinsons Dis..

[18] Zhang M.Y., Katzman R., Salmon D., Jin H., Cai G.J., Wang Z.Y., Qu G.Y., Grant I., Yu E., Levy P. (1990). The Prevalence of Dementia and Alzheimer’s Disease in Shanghai, China: Impact of Age, Gender, and Education. Ann. Neurol..

[19] Martinez-Horta S., Kulisevsky J. (2019). Mild Cognitive Impairment in Parkinson’s Disease. J. Neural. Transm..

[20] Williams-Gray C.H., Evans J.R., Goris A., Foltynie T., Ban M., Robbins T.W., Brayne C., Kolachana B.S., Weinberger D.R., Sawcer S.J. (2009). The Distinct Cognitive Syndromes of Parkinson’s Disease: 5 Year Follow-up of the CamPaIGN Cohort. Brain.

[21] Muslimovic D., Post B., Speelman J.D., Schmand B. (2005). Cognitive Profile of Patients with Newly Diagnosed Parkinson Disease. Neurology.

[22] Chung S.J., Lee H.S., Kim H.-R., Yoo H.S., Lee Y.H., Jung J.H., Baik K., Ye B.S., Sohn Y.H., Lee P.H. (2020). Factor Analysis-Derived Cognitive Profile Predicting Early Dementia Conversion in PD. Neurology.

[23] Zhu K., van Hilten J.J., Marinus J. (2014). Predictors of Dementia in Parkinson’s Disease; Findings from a 5-Year Prospective Study Using the SCOPA-COG. Parkinsonism Relat. Disord..

[24] Schrag A., Siddiqui U.F., Anastasiou Z., Weintraub D., Schott J.M. (2017). Clinical Variables and Biomarkers in Prediction of Cognitive Impairment in Patients with Newly Diagnosed Parkinson’s Disease: A Cohort Study. Lancet Neurol..

[25] Guo Y., Liu F.-T., Hou X.-H., Li J.-Q., Cao X.-P., Tan L., Wang J., Yu J.-T. (2020). Predictors of Cognitive Impairment in Parkinson’s Disease: A Systematic Review and Meta-Analysis of Prospective Cohort Studies. J Neurol..

[26] Levy G., Schupf N., Tang M.-X., Cote L.J., Louis E.D., Mejia H., Stern Y., Marder K. (2002). Combined Effect of Age and Severity on the Risk of Dementia in Parkinson’s Disease. Ann. Neurol..

[27] Nie K., Zhang Y., Wang L., Zhao J., Huang Z., Gan R., Li S., Wang L. (2012). A Pilot Study of Psychometric Properties of the Beijing Version of Montreal Cognitive Assessment in Patients with Idiopathic Parkinson’s Disease in China. J. Clin. Neurosci..

[28] Mao J., Huang X., Yu J., Chen L., Huang Y., Tang B., Guo J. (2020). Association Between REM Sleep Behavior Disorder and Cognitive Dysfunctions in Parkinson’s Disease: A Systematic Review and Meta-Analysis of Observational Studies. Front. Neurol..

[29] Jozwiak N., Postuma R.B., Montplaisir J., Latreille V., Panisset M., Chouinard S., Bourgouin P.-A., Gagnon J.-F. (2017). REM Sleep Behavior Disorder and Cognitive Impairment in Parkinson’s Disease. Sleep.

[30] Kotagal V., Albin R.L., Müller M.L.T.M., Koeppe R.A., Chervin R.D., Frey K.A., Bohnen N.I. (2012). Symptoms of Rapid Eye Movement Sleep Behavior Disorder Are Associated with Cholinergic Denervation in Parkinson Disease. Ann. Neurol..

