# Supplementary material for: Application of the Chinese Version of the Montreal Cognitive Assessment-Basic for Assessing Mild Cognitive Impairment in Parkinson’s Disease
Source: Brain Sci. 2021 Nov 28;11(12):1575. doi: 10.3390/brainsci11121575 (PMC8699542; doi:10.3390/brainsci11121575)
Supplement: Supplementary file 1 [file brainsci-11-01575-s001.zip › brainsci-1432983-supplementary.pdf]

Table S1. Drug usage of enrolled patients with PD

**Items**	**Total PD**	**PD-NC**	**PD-MCI**	***P value***
Without medication, *n*(%)	74(35.7%)	47(34.3%)	27(38.6%)	0.297
Levodopa, *n*(%)	123(59.4%)	82(59.9%)	41(58.6%)	0.859
Dopamine agonist, *n*(%)	81(39.1%)	55(40.1%)	26(37.1%)	0.675
MAO-BI, *n*(%)	18(8.7%)	15(10.9%)	3(4.3%)	0.107
COMTI, *n*(%)	8(3.9%)	5(3.6%)	3(4.3%)	1.000
Amantadine, *n*(%)	10(4.8%)	5(3.6%)	5(7.1%)	0.444
Artane, *n*(%)	3(1.4%)	1(0.1%)	2(2.9%)	0.551
LEDD (mg/d)	225.0(0.0,400.0)	225.0(0,375.0)	168.8(0,400.0）	0.836

Abbreviations: PD, Parkinson’s disease; PD-NC, Parkinson’s disease with normal cognition; PD-MCI, Parkinson’s disease with mild cognitive impairment; MAO-BI, monoamine oxidase-B inhibitor; COMTI, Catechol O-methyltransferase inhibitor; LEDD, Levodopa equipment daily dose.
